# GLAPD: Whole Genome Based LAMP Primer Design for a Set of Target Genomes

**DOI:** 10.3389/fmicb.2019.02860

**Published:** 2019-12-13

**Authors:** Ben Jia, Xueling Li, Wei Liu, Changde Lu, Xiaoting Lu, Liangxiao Ma, Yuan-Yuan Li, Chaochun Wei

**Affiliations:** ^1^Department of Bioinformatics and Biostatistics, School of Life Sciences and Biotechnology, Shanghai Jiao Tong University, Shanghai, China; ^2^Shanghai Center for Bioinformation Technology, Shanghai, China

**Keywords:** LAMP, group-specific primer, whole genome based primer designer, foodborne pathogens, primer design

## Abstract

Loop-mediated isothermal amplification (LAMP) technology has been applied in a wide range of fields such as detection of foodborne bacteria and clinical pathogens due to its simplicity and efficiency. However, existing LAMP primer designing systems require a conserved gene or a short genome region as input, and they can’t design group-specific primers. With the growing number of whole genomes available, it is possible to design better primers to target a set of genomes with high specificity based on whole genomes. We present here a whole Genome based LAMP primer designer (GLAPD), a new system to design LAMP primer for a set of target genomes using whole genomes. Candidate single primer regions are identified genome wide and then combined into LAMP primer sets. For a given set of target genomes, only primer sets amplifying them and only these genomes will be output. In order to accelerate the primer designing, a GPU version is provided as well. The effectiveness of primers designed by GLAPD has been assessed for a wide range of foodborne bacteria. GLAPD can be accessed at http://cgm.sjtu.edu.cn/GLAPD/ or https://github.com/jiqingxiaoxi/GLAPD.git. A simple online version is also supplied to help users to learn and test GLAPD: http://cgm.sjtu.edu.cn/GLAPD/online/.

## Introduction

Loop-mediated isothermal amplification (LAMP) is a simple-operating, effective and reliable method to amplify DNA sequence (Notomi et al., 2000; Parida et al., 2008; Mori and Notomi, 2009). The amplification is under a constant temperature (about 62°C) and the running time is short (within 1 h). In many application scenarios, LAMP is a better option than polymerase chain reaction (PCR) because the reaction can be in small and portable devices (Curtis et al., 2012; Chaumpluk et al., 2016). A basic LAMP primer set contains four synthetic primers derived from six primer regions (Supplementary Figure S1). Therefore, LAMP primer design is more complex than PCR primers. Designing a LAMP primer set to specifically identify a group of genomes (group-specific) at the same time has a high demand in many application fields like foodborne harmful bacteria detection, clinical pathogen identification, agricultural pathogen identification, and so on.

The group-specific primers mean that they can be applied to many target genomes belonging to a group (the primers are common) and at the same time the primers can’t amplify any other genomes not included in this group (the primers are specific) (Jarman, 2004; Kalendar et al., 2017). For example, there are 16 strains of white spot syndrome virus with complete genomes and more than 100 thousand other viruses in NCBI nucleotide database (up to October 29th, 2018). A group-specific primer for white spot syndrome virus should only amplify the 16 strains but no other viruses. Traditionally, the group-specific primers are designed based on conserved genes (Peng et al., 2015), genome regions (Yao et al., 2016) or the multiple sequence alignment (MSA) of these genes or genomic regions (Kurosaki et al., 2010). But this method is limited by the small number of suitable genes and the difficulty to generate MSA for a large number of sequences (Chen and Tompa, 2010). In addition, the primers based on traditional methods often can’t meet the requirements in practice. For example, the LAMP primer set from Wang et al. (2015) targeting *Staphylococcus aureus* was not able to amplify some *S. aureus* strains and might amplify some unexpected genomes (more details in results part). With more whole genomes available (O’Leary et al., 2016), it’s a better method to design group-specific primers based on whole genomes (Treven, 2015; Demkin et al., 2017).

There are some systems for LAMP primer design now. The most popular one is PrimerExplorer V5^1^, an online software. However, the maximum length of its input sequence is limited up to 2,000 bps. Therefore, designing primers based on the whole genome is not provided. In addition, common or specific primers can be designed by PrimerExplorer V5 using MSA results by clicking the “Common” or “Specific” button separately. However, these two buttons can’t work at the same time, which makes it not straightforward to design group-specific primers. Another system is LAVA (Torres et al., 2011), which can design common primers for a group of target genomes. However, it requires MSA results as the input, which limits the target region to a gene or conserved genomic region. LAVA doesn’t check the specificity of primers. FastPCR (Kalendar et al., 2017) is a system that can design LAMP primers using the whole genome. Similar as PrimerExplorer, it has an online version, and it designs common primers or specific primers separately. It can’t design group-specific primers in one run. According to the authors’ best knowledge, no existing system can design group-specific LAMP primers using whole genomes.

Here we present GLAPD (whole genome based LAMP primer designer), a new system to design group-specific LAMP primer sets. By using the whole genome sequences as input data, GLAPD can ensure the specificity of the primers and increase the chance to design a successful primer set. A graphics processing unit (GPU) version of GLAPD is also provided.

## Materials and Methods

### The Genome Based LAMP Primer Design System GLAPD

**FIGURE 1:**
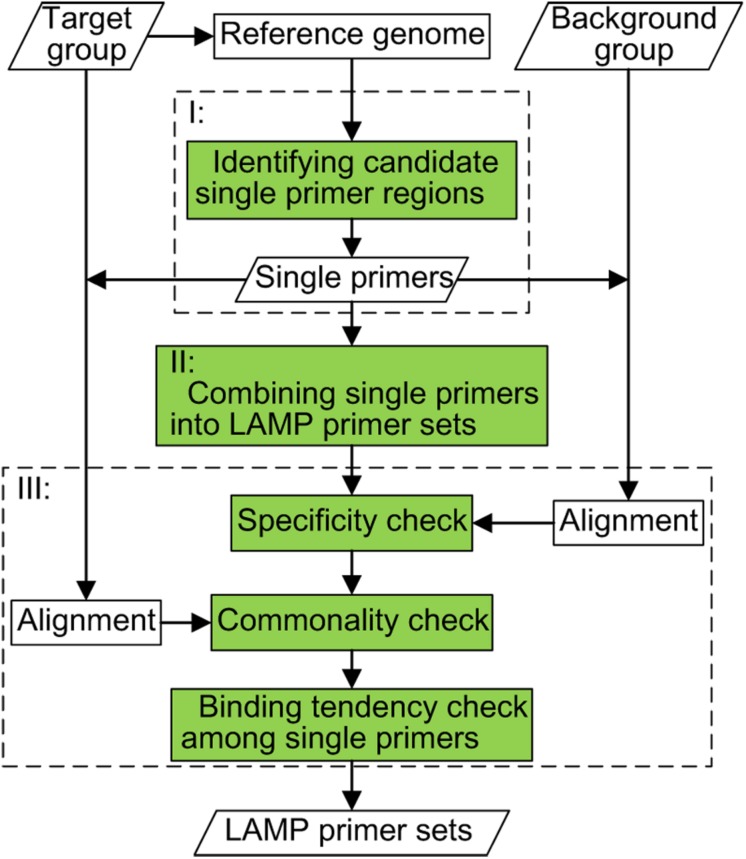
The system diagram of GLAPD. The inputs of GLAPD include the target group of genomes, the reference genomes, and the background group of genomes. There are three steps to generate LAMP primer sets: (I) identifying candidate single primer regions; (II) combining single primers into LAMP primer sets; (III) checking the LAMP primer sets. The check part contains commonality check based on target group, specificity check based on the background group and the tendency check of binding among single primers. Parts I, II, and III could be accelerated by using GPU.

The system diagram of GLAPD is listed in Figure 1. GLAPD has three steps: (I) identifying candidate single primer regions; (II) combining single primers into LAMP primer set; and (III) checking the LAMP primer set. The inputs, outputs and the computation steps are listed below in more details.

#### Inputs

In this step, two groups are defined first. The target group is defined as a group of genomes or genome regions which are expected to be amplified by the LAMP primer set. The primers generated by GLAPD are expected to identify each target genome. If it failed to generate a primer set for the group of target genomes, the system will output primers that can amplify the maximum number of genomes.

Similar to the target group, a background group is defined as a group of genomes or genome regions which are not expected to be amplified. Primers designed by GLAPD should not amplify any genome in the background group.

One genome from the target group needs to be selected as the reference genome which will be used as the temperate to generate primer sequences. The reference genome can be randomly picked from the target group.

#### Identifying Single Primer Regions

A basic LAMP primer set contains four synthetic primers from six primer regions, named F3, F2, F1c, B1c, B2, and B3. Sequences from F1c and F2 are synthesized into one primer FIP and sequences from B1c and B2 are synthesized into another primer BIP. The positions relationship among these single primers are showed in Supplementary Figure S1. In order to design a LAMP primer set, those candidate primer regions are identified first. They are then combined into LAMP primer sets.

Genome based LAMP primer designer identifies all candidate primer regions in the reference genome according to primer’s length, GC-content, melting temperature (Tm), stability and so on (Supplementary Table S1). The secondary structure of primer is checked by GLAPD using the thermodynamical approach similar to Primer3 program (Untergasser et al., 2012). At the same time, the end of each primer is checked to exclude symmetric sequences and homopolymers. GLAPD uses customized parameters to identify primer regions according to the GC-content of the target region (between F3 and B3). If GC-content of the target region is high, the GC-content and Tm of primers are set to be high, vice versa.

#### Combining Single Primers Into Primer Sets

Primers from six regions are combined into one basic LAMP primer set. GLAPD uses the positional relationship (Supplementary Figure S1) among the six regions, GC-content relationship between primers and whole amplification region (Supplementary Table S1), Tm relationship among primers (the Tms of F1c and B1c are 3°C higher than other primers’) to combine them. Then the combined LAMP primer set will be checked for commonality, specificity and tendency of binding among single primers.

#### Checking Commonality and Specificity of the LAMP Primer Set

An ideal LAMP primer set should be able to amplify all the target genomes but not genomes from the background group. In order to do this check, firstly, all single primers are aligned to the target genomes and the background genomes using Bowtie (Langmead et al., 2009). By default, no mismatch is allowed when a primer is aligned to the target group. If GLAPD fails to design LAMP primer sets to amplify all target genomes, a small number of mismatches are allowed when primers are aligned to the target group. However, if a primer can be aligned to a background genome within two mismatches (by default), this primer is considered as not specific. The more mismatches required to align the primer to the background genomes, the more specific is the primer. No matter how many mismatches in a primer, no mismatch is allowed in the 5′ of F1c and B1c, and the 3′ of F3, F2, B2, B3, LF, and LB primer. After the alignment, primers’ positions, strand information (plus or minus) and the number of mismatches in each genome are recorded.

Using the information generated above and the positional relationship of the six primer regions (Supplementary Figure S1), GLAPD checks the ability of a LAMP primer set to amplify genomes in the background group. If a LAMP primer set can amplify any background genome, this set will be discarded. Therefore, more flexible thresholds for positional relationship among primers can be used to improve the specificity in this step. After the LAMP primer set passes this specificity check, the number of genomes or genome regions in the target group amplified by the primer set is calculated using the same method in specificity check.

#### Checking the Binding Tendency of Any Two Primers

The LAMP primer set passed the commonality and specificity check will be checked for every single primer’s tendency of binding to other single primers in this primer set. This check uses the thermodynamical approach similar as the Primer3 program does.

#### Outputting LAMP Primer Sets

The LAMP primer set passed all above check steps will be output. When GLAPD has designed 10 (by default) LAMP primer sets successfully, or GLAPD has checked all candidate LAMP primer sets, the system stops automatically. The outputs contain the sequences, positions, lengths of the primers and genomes which can be amplified. The LAMP primer sets are not overlapped with each other. By default, the shortest distance between two LAMP primer sets’ F3 regions is 300 bps.

#### Loop Primers

In order to accelerate the amplification, two additional loop primers (LF and LB) can be added (Supplementary Figure S1). GLAPD can also design LAMP primer set with loop primers. The candidate primer regions are identified for loop primers from the reference genome first. Those candidate regions must meet the requirements listed in Supplementary Table S1. Then, GLAPD combines loop primers with other single primers into a LAMP primer set. A LAMP primer set could contain one or two loop primers. The Tms of loop primers are set to be 3°C higher than Tm of F3, F2, B2, and B3. At last, this LAMP primer set is checked for the tendency of primer annealing and its commonality.

### GPU Version

Graphics processing units can be used to accelerate GLAPD in three steps. In the step of identifying candidate single primer regions, GLAPD can identifies them from many positions of the reference genome simultaneously. Many primers’ GC-content, stability, Tm and secondary structure can be calculated in parallel in GPUs. In the step of combining single primers, each thread of GPU is assigned with a different candidate F3 primer then GLAPD tries to design all LAMP primer sets containing this F3 primer in parallel. In the checking step, each thread calculates the number of target genomes and background genomes that can be amplified by the primer set designed in this thread, and every single primer’s tendency of binding to other single primers. In each thread, only the LAMP primer set amplifying the maximum number of target genomes will be returned to CPU for output.

### Databases

Three databases were generated. Database-1 was the database of complete genome sequences of bacteria and archaea, which was downloaded from NCBI’s FTP on August 5th, 2013. It contained 4,902 sequence files from 2,599 strains (about 9GB).

Database-2 was the database of all complete mitochondrion sequences of suina, bovinae, and caprinae, which was downloaded from NCBI nucleotide database on June 14th, 2016. In this database, 120, 394, and 209 mitochondrion sequences for suina, bovinae and caprinae, respectively were selected.

Database-3 was the database of complete genome sequences of bacteria and archaea downloaded from NCBI’s Nucleotide database on September 19th, 2018. It contained 15,728 bacterial sequence files and 494 archaea sequence files (about 60GB).

Database-4 was the database of complete genome sequences of viruses downloaded from NCBI’s Nucleotide database on August 2th, 2017. It contained 163,576 viruses sequence files.

### DNA Extraction

The activated strains were cultured in appropriate method (Supplementary Table S2) and collected when the cultures reached an optical density at 600 nm (OD600) between 0.6∼1.0. The genomic DNA of the strains were extracted using AxyPrep^TM^ Multisource Genomic DNA Miniprep Kit (Axygen Bioscientific, Inc., United States). The samples were ground into powder with liquid nitrogen and homogenized with 400 μl of cell lysis buffer, and all other steps followed the manufacturer’ s instructions.

### LAMP Reaction

Loop-mediated isothermal amplification reaction was performed in a 25 μl reaction mixture. The mixture contained 1 × ThermoPol Buffer (contained 2 mmol/L MgSO4), 6 mmol/L MgSO4 (total 8 mmol/L), 1.4 mmol/L of each dNTP, 1.6 μmol/L of each inner primers (FIP and BIP), 0.2μmol/L of each outer primers (F3 and B3), 8 units of Bst DNA Polymerase (Large Fragment).

When test the LAMP primer designed for *S. aureus*, the mixture contained 20 ng of template DNA of each 29 bacterial strains. When test the LAMP primer’s specificity designed for *Vibrio vulnificus* and *Vibrio cholerae*, the mixture also contained 20 ng of template DNA of each 29 bacterial strains and when test its commonality, the amount of template DNA was 50 ng, 5 ng, 500 pg, 50 pg, 5 pg, 500 fg, 50 fg, 5 fg and 0.5 fg, respectively. In all NTC (no template control) reaction, template DNA was replaced by sterilized water.

The LAMP reaction was carried out at 62°C for 60 min using a Veriti^TM^ Dx Thermal Cycler (Thermo Fisher, United States), then inactivated Bst DNA Polymerase at 80°C for 10 min. After the reaction, 1 μl 1000× SYBR Green I was added into the solution to confirm whether the reaction occurred. In a positive reaction, the color of the solution was green and in a negative reaction, the color was orange.

## Results

### Experimental Validation of the Group-Specific Primers Designed by GLAPD

#### Group-Specific LAMP Primers of *Staphylococcus aureus*

*Staphylococcus aureus* is one main type of foodborne pathogens around the world (Kadariya et al., 2014; Paudyal et al., 2017). Traditionally, its *nuc*, *mecA* genes were used to design the LAMP primer sets (Wang et al., 2015; Chen et al., 2017). But the two genes are not conserved among all *S. aureus* (Hoegh et al., 2014; Karmakar et al., 2016) and may exist in other *Staphylococcus* spp. (Borjesson et al., 2015). Therefore, existing LAMP primer sets (Wang et al., 2015) for *S. aureus* are neither common nor specific enough (Supplementary Tables S3, S4).

In database-1 the *S. aureus* species had 43 strains (Supplementary Table S3). GLAPD used those 43 strains as the target group and the rest of the genomes in database-1 as the background group then designed several group-specific LAMP primer sets. One set (Table 1) was located in a predicted gene which codes 50S ribosomal protein L14. This LAMP primer set was common for all *S. aureus* and not for any other bacteria. It was validated by experiments (Supplementary Figure S2).

**TABLE 1:** The LAMP primer set designed by GLAPD for *S. aureus*.

**Primer**	**Sequence (5′-3′)**
F3	GTCATTACGACGAACACC
B3	ATCCAACAAGAAACACGC
FIP	TTAAAAATGCAACACCAGGTGG-CTTAGTACGTACGATTACAGC
BIP	GCTGTTTTACGACCAGATCCA-TGAAAGTAGCAGACAACTCT

#### *Vibrio* Group-Specific LAMP Primer Set

*Vibrio* spp. is one of the main pathogenic bacteria in seafood (Huehn et al., 2014; Mizan et al., 2015). Most LAMP primers are designed only for the identification of *V. cholerae* (Okada et al., 2010) or *V. vulnificus* (Han et al., 2011). In spite that the *V. vulnificus* and *Vibrio parahaemolyticus* can be detected simultaneously in one reaction, it required two LAMP primer sets in the reaction (Wang et al., 2016).

In database-1, there were eight strains of *V. cholerae* and three strains of *V. vulnificus* (Supplementary Table S5). *V. cholerae* and *V. vulnificus* belonged to *Vibrio* genus. GLAPD used those 11 strains as the target group and the rest of the genomes in database-1 as the background group. Several group-specific LAMP primer sets were designed by GLAPD. One set (Table 2) was located in a gene which coded 30S ribosomal protein S20. This LAMP primer set was common for all *V. cholerae* and *V. vulnificus*, and was specific to these bacteria only. It was validated by experiments listed in Supplementary Figure S3.

**TABLE 2:** The LAMP primer set designed by GLAPD for *V. cholerae* and *V. vulnificus*.

**Primer**	**Sequence (5′-3′)**
F3	CGAGACTTGTGACGAGCTG
B3	TCCAAGCTGAGAAACGTCG
FIP	TGCAACTGCTGCATTCGCTGTA-TAAGGCCTTTAGTCGCCATG
BIP	CCAGCAGCGATTGCAGCAAC-ACAATGCTAGCCGTCGTTC

Besides the two group-specific LAMP primer sets mentioned above, we have applied GLAPD to design group-specific primers for more than ten other foodborne pathogens. GLADP could successfully design group-specific primers for most of these foodborne pathogens and more than half of those primers worked well in real experiments (Supplementary Table S6).

The flexible setting of target and background group.

When GLAPD is used to design group-specific primers, the target group and background group could be defined flexibly by users. Some examples are listed below to show this flexibility.

(1) To design group-specific primer for all genomes in a genus. *Salmonella* is a common bacterial pathogen responsible for salmonellosis, a common disease affects the intestinal trace and it can cause substantial socioeconomic burden (Liu et al., 2018). In database-3, 471 complete genomes and 1 assembly genome with 66 contigs were *Salmonella*. GLAPD could design the genus-level salmonella-specific LAMP primer set successfully. One LAMP primer set designed by GLAPD (Supplementary Table S7) was in a gene coding (2E,6E)-farnesyl diphosphate synthase. It could amplify all 472 genomes without any mismatches, and it was specific to all target genomes considering all other genomes in database-3 as the background genomes.

(2) To design group-specific primer for some genomes in a genus. Both *V. cholerae* species and *V. vulnificus* species belong to *Vibrio* genus. In database-3, 44 complete genomes were *V. cholerae* and 19 genomes were *V. vulnificus*. The primer set listed in Table 2 was still common in all 63 genomes without any mismatches, and is specific to the target genome considering the other genomes in database-3 as the background group (don’t allow any mismatches).

(3) To design group-species primer for all genomes in a species. In database-3 there were 351 complete genomes of *S. aureus*, the primer set in Table 1 was neither common nor specific in the new database. One new primer set (Supplementary Table S8) designed based on database-3 was overlap with two genes coding S4 domain-containing protein *YaaA* and DNA replication repair protein *RecF*. It was common for all 351 genomes without any mismatches and specific to the target genomes as well.

(4) To design group-specific primer for some genomes in a species. Enterohemorrhagic *Escherichia coli O157*:*H7* was a major foodborne pathogen and it caused diarrhea (Tarr et al., 2005; Lim et al., 2010). The *E. coli O157*:*H7* was one group of *E. coli* species. In database-3, there were 59 complete genomes of *E. coli* and three were *O157*:*H7* (In order to exclude ambiguous group, only take care of part *E. coli* genomes, those accession number must start with “NC”). One LAMP primer set (Supplementary Table S9) designed by GLAPD overlaps with two genes coding recombinase and peptide transporter. This primer set was common for all three genomes without any mismatch and specific to all these three genomes as well (with all other genomes in database-3 as the background group).

### GLAPD Can Design Primers for Other Organisms

Genome based LAMP primer designer was first developed for rapidly detecting foodborne pathogens using LAMP technology. But group-specific primers for other organisms can also be designed by GLAPD. For example, we have tried to design group-specific primers to detect halal products using GLAPD. Pork is not allowed in halal products (Nakyinsige et al., 2012). The previous LAMP primer sets for pork identification were designed on *DN1* gene and *cytb* gene located in mitochondria (Yang et al., 2014; Ran et al., 2016). But the LAMP primer set (Yang et al., 2014) couldn’t cover all mitochondria genomes (Supplementary Table S10). In database-2, there were 120 mitochondria sequences of sunia which were considered to be from pork products. We used GLAPD to design several LAMP primer sets with the 120 sunia mitochondrion sequences as the target group and the rest in database-2 as the background group. One of these primer sets (Supplementary Table S11) was in a 16S rRNA gene. This LAMP primer set was common for all pork mitochondria and would amplify the pork mitochondria only.

We also tried to design group-specific primers for several types of aquatic animal viruses in database-4. GLAPD could design group-specific primers for most aquatic animal viruses using default parameters (Supplementary Table S12). The group-specific primers of infectious hematopoietic necrosis virus and spring viraemia of carp virus were validated by experiments. If GLAPD couldn’t design group-specific primers, it would output the most common primers.

### Comparison With Existing Systems

**FIGURE 2:**
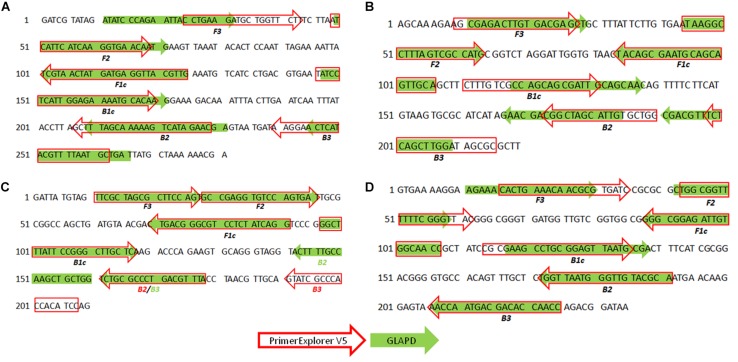
Comparison of the primer sets designed by GLAPD and PrimerExplorer V5. The sequence containing LAMP primer set designed by GLAPD was input to PrimerExplorer V5 to design primers. Four scenarios of LAMP primer sets were shown: **(A)** LAMP primer set for *S. aureus* based on database-3; **(B)** LAMP primer set for *V. cholerae* and *V. vulnificus*; **(C)** LAMP primer set for *Salmonella* based on database-3; **(D)** LAMP primer set for *E. coli O157*:*H7* based on database-3. The primers designed by GLAPD were shown in green solid arrows and primers designed by PrimerExplorer V5 were in red blank arrows. The *Salmonella*’s B2 primer designed by PrimerExplorer was the same as the B3 primer designed by GLAPD.

PrimerExplorer V5 was an online LAMP primer design software. A candidate genomic region was required as the input. However, GLAPD did not require the prior knowledge about the candidate gene or genomic region. Using the same sequences from the candidate regions containing primer sets designed by GLAPD, the results of PrimerExplorer V5 and GLAPD were very similar. Most primers from two designers overlapped and some of them were identical (Figure 2). The main reason of differences was the different primer combination strategies.

**FIGURE 3:**
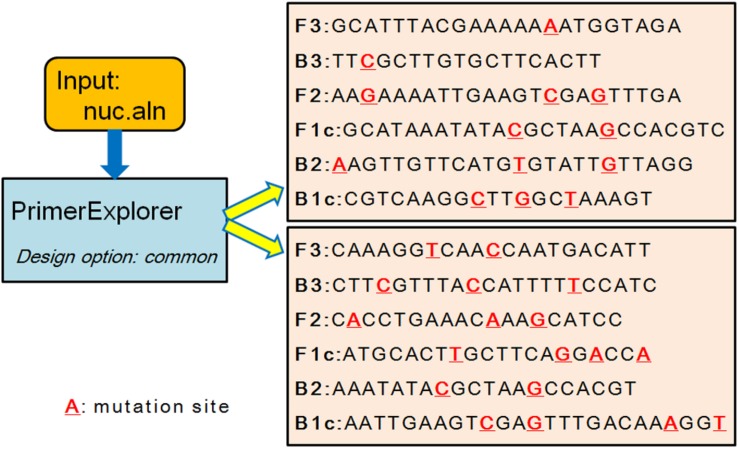
The common primer sets of *nuc* gene designed by PrimerExplorer. The two primer sets are design by PrimerExplorer using MSA result of non-redundant *nuc* gene sequences. The red nucleotide with red underline means a mutation among those gene sequences.

Existing systems such as PrimerExplorer, LAVA and FastPCR can design common primer set for a group of genomes without considering the specificity comparing to a background group (LAVA was not used to design primers because it was not downloadable anymore). Users can input a set of genomic regions considering the first region as the target and the rest as the background group to design specific primers. However, it is not straightforward to design primer sets directly with a given target group and a given background group at the same time. For example, the *mecA* and *nuc* genes were used as candidate regions (Wang et al., 2015; Chen et al., 2017) to design primers for *S. aureus*. In database-3, there were 351 *S. aureus* genomes, among which 235 genomes had *mecA* gene and all 351 genomes had *nuc* gene. The non-redundant 19 *mecA* sequences and 44 *nuc* sequences were used as inputs for PrimerExplorer and FastPCR to design primers. For *mecA* gene, the MSA result was generated by clustalW (Thompson et al., 1994) with default parameter, and only 68.1% nucleotides were identical. PrimerExplorer couldn’t design any LAMP primer sets using this MSA result and FastPCR also couldn’t design any primers using the 19 *mecA* sequences. For *nuc* gene, the MSA alignment result was also generated by clustalW and only 74.7% nucleotides were identical. FastPCR couldn’t design any common primers for the 44 sequences. PrimerExplorer could design two LAMP primer sets. However, because of the low similarity, there were many mutations in the designed primer regions in different target genomes (Figure 3, red underline), which indicated that the commonality of this primer set was not good. Therefore, using PrimerExplorer and FastPCR to design common primer set for *S. aureus* with the *mecA* and *nuc* gene regions as input was not successful. On the contrary, GLAPD could use the whole genome to design LAMP primer sets for the *S. aureus* genomes successfully. More examples are in Supplementary Material (Supplementary Figure S4 and Supplementary Table S14).

More comparisons between GLAPD and those existing systems were listed in Table 3.

**TABLE 3:** The comparison with existing LAMP primer designing systems.

	**GLAPD**	**PrimerExplorer**	**LAVA**	**FastPCR**
Input	Sequence	Sequence/MSA	Sequence/MSA	Sequence/MSA
Input length	Not limited	≤2,000 bps	Not limited	Not limited
common primers	Yes	Yes	Yes	Yes
specific primers	Yes	Yes	No	Yes
Group-specific	Yes	No	No	No
Graphical interface	No	Yes	No	Yes
Operating system	Unix/Linux	Windows	Unix/Linux	Windows
Version	Stand-alone	Online	Stand-alone	Online/Stand-alone
Language	C,CUDA C,Perl	Java	Perl	Java
License	GPL2	Free	BSD	Online: free Stand-alone: charge

## Discussion

### Why Do We Use Group-Specific Primers?

Detecting multiple foodborne pathogens simultaneously can help the food safety because it can speed the detection of pathogens. When use LAMP or PCR, the traditional method for this aim is adding multiple sets of primers into one assay. Each primer set can only be applied to one pathogen. This method has some disadvantages: (1) a large number of different primers in one assay may increase the risk of generating primer dimer, and the efficiency of one primer set could be inhibited by other primer sets (Xu et al., 2012; Zhao et al., 2014); (2) It’s a challenge task to find a feasible combination of multiple primer sets due to the huge number of different combinations of candidate primer sets in addition to the test of each primer set for each target genome.

Genome based LAMP primer designer, on the other hand, can design group-specific primers, which can avoid those disadvantages. In one assay, only one primer set is needed. Less primers in assay can decrease the risk of interactions among primers and reduce the test workload as well.

### Designing Primers Based on the Whole Genome

Traditionally, primers are designed for some conserved genes or a small genome region. Most regions of the whole genome are neglected. In addition, many primer design systems have a limited length of input sequences. If no primers could be designed by GLAPD, the chance to find suitable primers would be low.

Genome based LAMP primer designer can use whole genomes as input directly. It scans all candidate single primers derived from the whole genome, then all candidate primers are combined into primer sets and tested one by one. This can vastly improve the success rate of primer design.

### A New Strategy Is Used to Design Group-Specific Primers

The group-specific primers can be designed based on conserved genes, genomic regions or MSA results. However, existing methods have limitations: (1) There are limited number of well-known conserved genes in each organism. This number will be much smaller for a slightly larger number of different target organisms. (2) The conserved genes may exist in some background organisms. For example, the 16S rRNA genes are conserved in many bacteria, therefore non-target organisms may also be amplified if they contain very similar 16S rRNA genes; (3) It is difficult to generate a MSA results from a big number of input sequences, and it is almost impossible to generate MSA for many genomes.

Genome based LAMP primer designer uses a different strategy to avoid those problems. Firstly, GLAPD searches all candidate primers genome wide, then the candidate single primers are aligned with target and background genomes. The alignment information about positions and strands is used to check primers’ commonality and specificity. The distance between two single primers can be different in each target genome as long as the distance is within the allowed range. The sequences between primers can also be different in different target genomes as long as the primer regions are conserved among target genomes. In other words, GLAPD can design group-specific primers in variable genome regions with a higher success rate. This strategy can also be used in designing primers for other amplifications, like PCR.

### The Parameter Configuration in GLAPD

Parameters and thresholds used in GLAPD were similar with PrimerExplorer V5. Good results were still achieved if GLAPD used loose thresholds. For example, when the 5′ stability of F1c or B1c was set to be bigger than -4 kcal/mol, the experimental results of the primers were still good. More experiments might be needed to decide better parameters or thresholds for a specific group of organisms.

### GPU Version of GLAPD

In order to accelerate the LAMP primer design, a GPU version of GLAPD was developed. The GPU version was very promising to accelerate the primer design procedure in identifying candidate single primer regions (GPU version is three time faster than CPU version) and combining single primers to a primer set may be slower than CPU version in some scenarios (Supplementary Table S13). We are currently working on it to improve the performance of the GPU version.

## Conclusion

Designing group-specific primers is a difficult task for amplifications like PCR, and it is even more challenging for LAMP due to the number of primers in LAMP primer set. Here we present a new LAMP primer designer, GLAPD, to design a LAMP primer set targeting on a group of genomes. Instead of using well-known gene regions, the whole genome could be used directly for primer design, which increased the success rate.

Genome based LAMP primer designer could be applied to design LAMP primers for the identification of any organisms without known regions as input. The results of GLAPD are similar to PrimerExplorer V5 when the same sequences are input. The effectiveness of GLAPD were validated in experiments. With GLAPD, the chance to successfully design a LAMP primer set to identify a group of organism is higher than before and it can be a good system to accelerating the application of LAMP technology in many fields such as food quarantine, epidemic disease surveillance and so on. GLAPD can be downloaded from http://cgm.sjtu.edu.cn/GLAPD/ or https://github.com/jiqingxiaoxi/GLAPD.git. Users can also learn and test GLAPD using the simple online version: http://cgm.sjtu.edu.cn/GLAPD/online/.

## Data Availability Statement

All datasets generated for this study are included in the article/Supplementary Material.

## Author Contributions

CW: conceptualization and design of the system. BJ and CW: system implementation. BJ and LM: system test. XLi, WL, CL, XLu, and Y-YL: experimental validation of designed LAMP primers. BJ, XLi, Y-YL, and CW: writing the manuscript. All authors have read and edited the manuscript.

## Conflict of Interest

The authors declare that the research was conducted in the absence of any commercial or financial relationships that could be construed as a potential conflict of interest.

## Footnotes

1 http://primerexplorer.jp/e/
