# Supplementary material for: GLAPD: Whole Genome Based LAMP Primer Design for a Set of Target Genomes
Source: Front Microbiol. 2019 Dec 13;10:2860. doi: 10.3389/fmicb.2019.02860 (PMC6923652; doi:10.3389/fmicb.2019.02860)
Supplement: Supplementary file 1 [file Data_Sheet_1.pdf]

Supplementary Materials

1 Supplementary Figures and Tables

This file contains 4 figures and 14 tables (Tables S1-S2, S4-S9, S11-S13 and S15~S17). Table S3, S10 and S14 in Excel format are listed separately.

1.1 Supplementary Figures

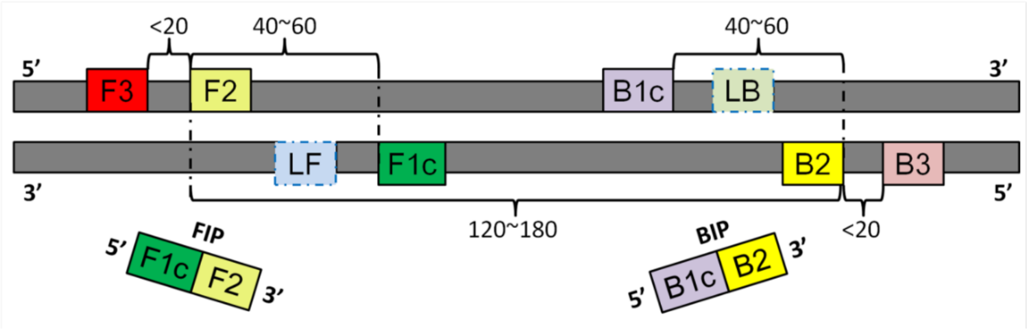

Figure S1. The positional relationship among primers in a LAMP primer set. A basic LAMP primer set contains four synthetic primers from six regions(F3, F2, F1c, B1c, B2 and B3, denoted by solid boxes). In order to accelerate the amplification, two additional loop primers(LF and LB, denoted by dotted boxes) can be added. The distance(bp) between two primers is marked on the brace.

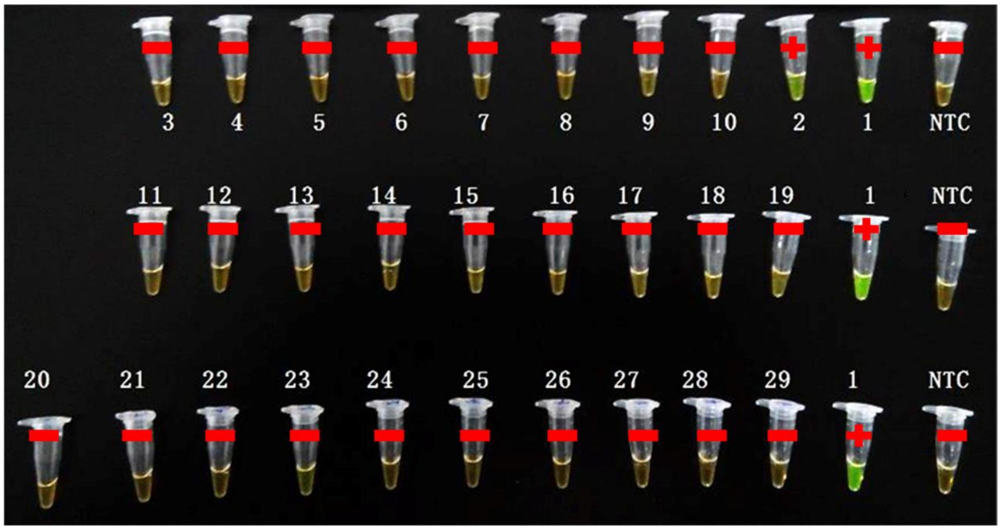

Figure S2. Validation of the LAMP primer set designed for *S. aureus*. The LAMP primer was applied to 29 bacterial strains: 1, *S. aureus*; 2, *S. aureus* subsp. *aureus*; and other strains that were not *S. aureus* (more details in Table S2). The tests were executed in three batches each of which contains a negative control without DNA template (labeled as NTCs). "+" means positive results and "-" stands for negative results.

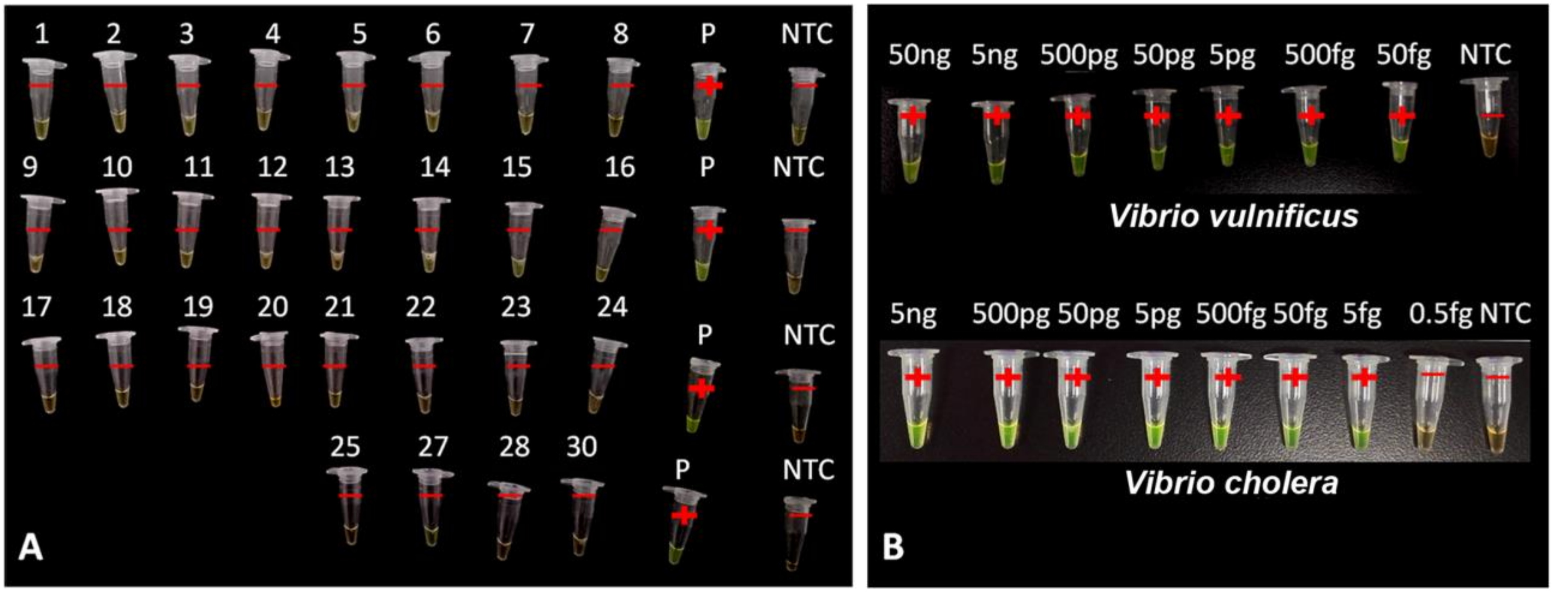

Figure S3. Validation of the LAMP primer set designed for *V. cholera* and *V. vulnificus*. A: Check the specificity of the LAMP primer set by applying it to 28 bacterial strains in addition to *V. cholera* and *V. vulnificu*s (more details in Table S2). B: The sensitivity of the LAMP primer set by applying different amount of DNA templates of *V. cholera* and *V. vulnificu*s. P stands for tests with DNA templates of V. *cholera* and V. *vulnificu*s. NTC stands for negative controls without template control. Label “+” means positive reactions and “-” stands for negative reactions.

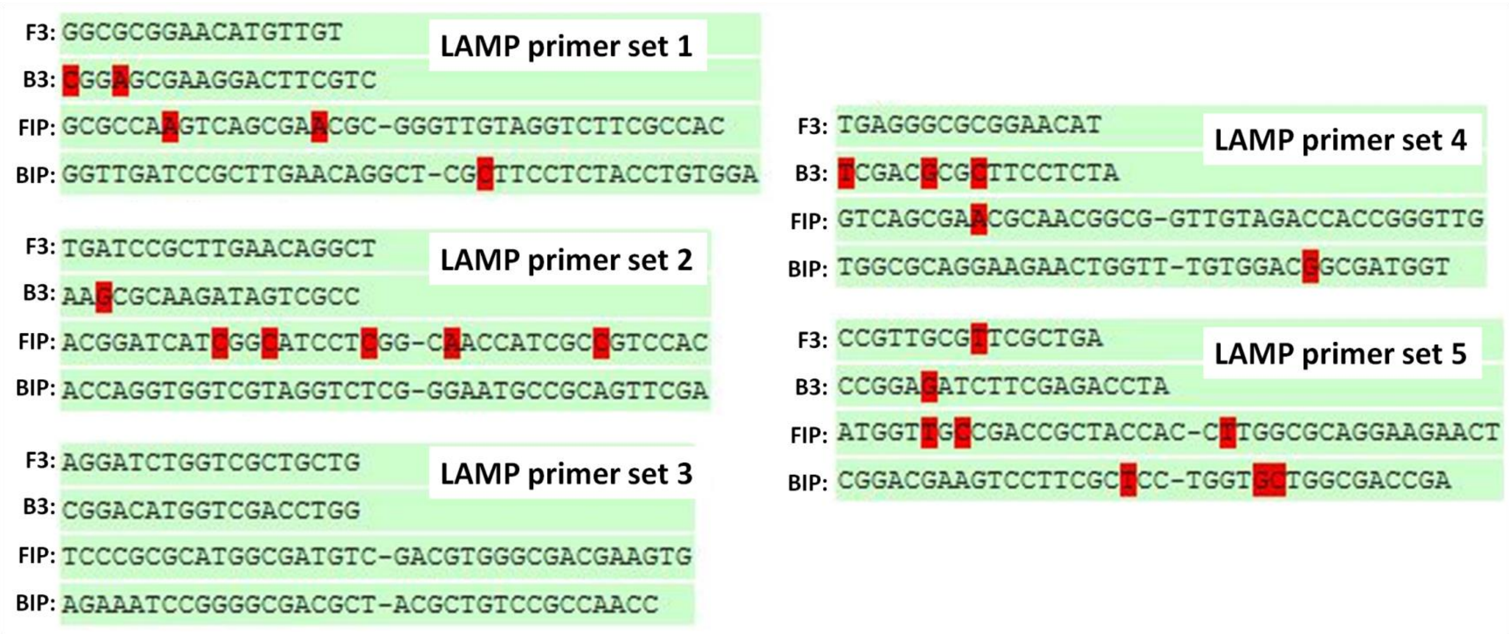

Figure S4. The common primer sets of *P. aeruginosa* designed by PrimerExplorer. The five primer sets are designed by PrimerExplorer using MSA result of *P. aeruginosa*-specific gene sequences. The nucleotides marked with red are mutations among those gene sequences.

1.2 Supplementary Tables

Table S1. Parameters for identifying candidate single primer regions.

**Primer region**	**Whole GC content**(%)[*1]	**Tm**(℃)	**Length** (bp)	**GC content** (%)	**5' ΔG** (kcal/mol)[*2]	**3' ΔG** (kcal/mol)
F3/F2/B3/B2	≤45	55~58	18~25	30~65	≤-3	≤-4
	≥60	59~63	15~20	40~70		
	45~60	59~61	18~20	40~65		
F1c/B1c	≤45	60~63	20~25	30~65	≤-4	≤-3
	≥60	64~68	15~22	40~70		
	45~60	64~66	20~22	40~65		
LF/LB	≤45	60~63	20~25	30~65	≤-3	≤-4
	≥60	64~68	15~22	40~70		
	45~60	64~66	20~22	40~65		

*1: the GC content is calculated based on the region from F3 to B3

*2: ΔG is for the change in free energy, and it is used to assess the stability of primers

Table S2. The source of 30 bacteria used in LAMP experiments.

No.	Name	Source	Catalog	Medium	Temp.
1	*Staphylococcus aureus*	CICC	21600	Nutrient agar	37℃
2	*Staphylococcus aureus* subsp. *aureus*	CGMCC	1.2465	Nutrient agar	37℃
3	*Staphylococcus epidermidis*	CGMCC	1.426	Nutrient agar	37℃
4	*Rhodococcus equi*	CGMCC	1.4262	Beef extract starch medium	37℃
5	*Bacillus cereus*	CGMCC	1.376	Nutrient agar	30℃
6	*Bacillus mycoides*	CICC	21473	Nutrient agar	30℃
7	*Listeria monocytogenes*	CICC	21635	Brain heart agar	37℃
8	*Listeria innocua*	CICC	10417	Brain heart agar	37℃
9	*Listeria ivanovii*	CICC	21663	Brain heart agar	37℃
10	*Salmonella enterica* subsp. enterica	CGMCC	1.1859	Nutrient agar	37℃
11	*Salmonella enterica* subsp. enterica serovar Enteritidis	CICC	21482	Nutrient agar	36℃
12	*Salmonella enterica* subsp. enterica serovar Typhimurium	CICC	10420	Nutrient agar	37℃
13	*Salmonella paratyphi* β	CICC	10437	Nutrient agar	37℃
14	*Shigella dysenteriae*	CGMCC	1.1869	Nutrient agar	37℃
15	*Shigella boydii*	CGMCC	1.10618	Nutrient agar	30℃
16	*Shigella flexneri*	CGMCC	1.1868	Nutrient agar	37℃
17	*Escherichia coli*	CICC	10783	LB medium	37℃
18	*Escherichia coli* EPEC 026:K60	CICC	10372	Nutrient agar	37℃
19	*Escherichia coli* EPEC O127:K63	CICC	10411	Nutrient agar	37℃
20	*Escherichia coli ETEC O126：K71*	CICC	10415	Nutrient agar	37℃
21	*Escherichia coli* ETEC	CICC	10665	Nutrient agar	37℃
22	*Escherichia coli* EHEC O157:H7	CICC	21530	Nutrient agar	36℃
23	*Enterobacter sakazakii*	CICC	21560	Nutrient agar	36℃
24	*Yersinia enterocolitica*	CICC	21669	Nutrient agar	26℃
25	*Yersinia pseudotuberculosis*	CMCC	53504	Hottinger agar	28℃
26	*Vibrio vulnificus*	CICC	21615	3.5% sodium chloride agar	30℃
27	*Vibrio parahaemolyticus*	CGMCC	1.1997	Hottinger agar	37℃
28	*Vibrio furnissii*	CGMCC	1.1613	Ammonium ferric citrate medium	30℃
29	*Vibrio cholerae*	CGMCC	1.8676	Ammonium ferric citrate medium	30℃
30	*Shigella sonnei*	CICC	21535	Nutrient agar	36℃

CICC: China Center of Industrial Culture Collection
CMCC: National Center for Medical Culture Collections
CGMCC: China General Microbiological Culture Collection Center

Table S4. Number of mismatches between existing LAMP primer sets with two non-*S. aureus*. Ten LAMP primer sets from Xinru Wang *et al.* (Rapid Detection of *Staphylococcus Aureus* by Loop-Mediated Isothermal Amplification) were aligned to all bacteria in databae-1 excluding 43 *S. aureus* strains (as the background group) using Bowtie with parameters "-v 2 -a". Five LAMP primer sets could align to two non-*S. aureus* bacteria and they could amplify the two unexpected bacteria. All mismatches were recorded in this table. The less mismatches, the less specificity of the LAMP primer set. Results showed that five LAMP primer sets were not enough specific for *S. aureus*.

**Primer set**	*Staphylococcus haemolyticus JCSC1435*(NC_007168.1)	*Staphylococcus epidermidis RP62A*(NC_002976.3)
mecA-1	1	1
mecA-2	0	0
mecA-3	0	0
mecA-4	0	0
mecA-5	0	0

Table S5. All *V. cholera* and *V. vulnificus* in database-1.

Accession	Name
NC_016944.1	*Vibrio cholerae* IEC224 chromosome I
NC_016945.1	*Vibrio cholerae* IEC224 chromosome II
NC_017269.1	*Vibrio cholerae* LMA3984-4 chromosome II
NC_017270.1	*Vibrio cholerae* LMA3984-4 chromosome chromosome I
NC_012578.1	*Vibrio cholerae* M66-2 chromosome I
NC_012580.1	*Vibrio cholerae* M66-2 chromosome II
NC_012667.1	*Vibrio cholerae* MJ-1236 chromosome 2, complete genome
NC_012668.1	*Vibrio cholerae* MJ-1236 chromosome 1
NC_016445.1	*Vibrio cholerae* O1 str. 2010EL-1786 chromosome 1
NC_016446.1	*Vibrio cholerae* O1 str. 2010EL-1786 chromosome 2
NC_002505.1	*Vibrio cholerae* O1 biovar El Tor str. N16961 chromosome I
NC_002506.1	*Vibrio cholerae* O1 biovar El Tor str. N16961 chromosome II
NC_012582.1	*Vibrio cholerae* O395 chromosome chromosome I
NC_012583.1	*Vibrio cholerae* O395 chromosome chromosome II
NC_009456.1	*Vibrio cholerae* O395 chromosome 1
NC_009457.1	*Vibrio cholerae* O395 chromosome 2
NC_004459.3	*Vibrio vulnificus* CMCP6 chromosome I
NC_004460.2	*Vibrio vulnificus* CMCP6 chromosome II
NC_014965.1	*Vibrio vulnificus* MO6-24/O chromosome I
NC_014966.1	*Vibrio vulnificus* MO6-24/O chromosome II
NC_005139.1	*Vibrio vulnificus* YJ016 chromosome I
NC_005140.1	*Vibrio vulnificus* YJ016 chromosome II

Table S6. The statistics of group-specific LAMP primer sets designed for foodborne pathogens. The group-specific primers are designed by GLAPD to amplify each genome in target group without any mismatches, and they are specific to each genome in background group even allowing two mismatches in each single primer.

**Foodborne pathogen**	**#Target genome**	**Background group**	**#LAMP primer set in test***	**#Success**
Escherichia coli O157:H7	4	others in db-1	8	1
Staphylococcus aureus	43	others in db-1	10	8
Listeria monocytogenes	37	others in db-1	9	9
Bacillus cereus	13	others in db-1	no group-specific primers	
Salmonella	38	others in db-1	10	6
Shigella	9	others in db-1	10	7
Yersinia enterocolitica	3	others in db-1	10	6
Yersinia pseudotuberculosis	4	others in db-1	10	1
Vibrio cholerae	8	others in db-1	10	8
Vibrio parahaemolyticus	4	others in db-1	10	9
Vibrio vulnificus	3	others in db-1	9	9
Cronobacter sakazakii	3	others in db-1	10	10
Escherichia coli ETEC	1	others in db-1	10	1
Clostridium botulinum	13	others in db-1	no group-specific primers	
Total	183		116	75

*: In test, if the LAMP primer set can amplify one target genome successfully and in the same time the negative control experiment without DNA template is negative result, the test is successful.

Table S7. The group-specific LAMP primer set designed for *Salmonella* based on database-3.

Primer	Sequence(5'-3')
**F3**	TTCGCTAGCGCTTCCAGT
**B3**	TAAACGTCAGGGCGCAGA
**FIP**	CCTGATAGAGGACGCCCGTCAG-GCCGAGGTGTCCAGTGAT
**BIP**	GGCTTTATTCCGGGCTTGCTCA-CCAGCAGCTTGGCAAAAGT

Table S8. The group-specific LAMP primer set designed for *S. aureus* based on database-3.

Primer	Sequence(5'-3')
**F3**	ATATCCCAGAATTACCTGAAGA
**B3**	ATCAGCATTAAAACGTATGAGT
**FIP**	CAACGTAACCTCATCATAGTTACGA-TCATTCATCAAGGTGAACAATG
**BIP**	ATCCTCATTGGAGAAAATGCACAAG-TCGTTCTATGACTTTTTGCTAAA

Table S9. The group-specific LAMP primer set designed for *E. coli* O157:H7 based on database-3.

Primer	Sequence(5'-3')
**F3**	AGAAACACTGAAACAACGCG
**B3**	GGTTGGTGTCGTCATTGGTT
**FIP**	GGTTGCCACAATCTCCGCCC-CTGGCGGTTTTTTCGGGT
**BIP**	GAAGCCTGCGGAGTTAATGCGA-TGCGTACAACCCATTAACCA

Table S11. The group-specific LAMP primer set designed for pig mitochondria.

Primer	Sequence(5'-3')
**F3**	GTTTACGACCTCGATGTTG
**B3**	TCTCTGAGACGCGTTTGT
**FIP**	CACGTAGGACTTTAATCGTTGAACA-GATCAGGACACCCAAATGG
**BIP**	GGAGCAATCCAGGTCGGTTT-GGTTGGTCCCATTTCTCT

Table S12. The statistics of group-specific LAMP primer sets designed for aquatic animal viruses. The group-specific primers are designed by GLAPD to amplify each genome in target group without any mismatches, and they are specific to each genome in background group even allowing two mismatches in each single primer.

Aquatic animal virus	#Target genome	Background group	Group-specific primer
Spring viraemia of carp virus	12	others in db-4	Yes*
White spot syndrome virus	10	others in db-4	Yes
Infectious hematopoietic necrosis virus	7	others in db-4	Yes
Cyprinid herpesvirus	14	others in db-4	Yes
Cyprinid herpesvirus 2	4	others in db-4	Yes
Grass carp reovirus	13	others in db-4	No
Infectious hypodermal and hematopoietic necrosis virus	28	others in db-4	Yes

*: One mismatch between single primer and target genomes is allowed.

Table S13. Speed comparison between the CPU version and GPU version (second). All primer designs are carried out on a CentOS 6.7 Linux machine with Xeon e7-4807 CPU, 64 GB memory and an NVIDIA Tesla C2050 GPU. The unit for computing time is second.

	**Reference**(accession)	NC_002951.2	KC469586.1
	**Target group**	43 *S. aureus*	120 suina mitochondrion
	**Background group**	others in db-1	others in db-2
**Identifying single primer regions**	CPU version	30,941	228
	GPU version	10,084	77
**Combining single primers into LAMP primer sets**	CPU version	1,799	108
	GPU version	28,680	2,100

Table S15. The group-specific LAMP primer set designed by GLAPD for *H. pylori* based on database-1.

Primer	Sequence(5'-3')
**F3**	GTAGGCGGGATAGTCAGT
**B3**	TAATGTTCCAGCAGGTCG
**FIP**	ACCTCTCCCACACTCTAGAATAGT-CAGGTGTGAAATCCTATGGC
**BIP**	AGGTGGAATTCTTGGTGTAGGG-CCTTCGCAATGAGTATTCCT

Table S16. Number of mismatches between an existing LAMP primer set and 9 *P. aeruginosa* strains. One LAMP primer set from Chao Li *et al.* was aligned to 9 *P. aeruginosa* strains (as target group) using Bowtie with the "-v 2 -a" parameters. The numbers of mismatches were shown in this table. The more mismatches, the less commonality of the LAMP primer set. Results showed that this LAMP primer set was not common enough to cover all target *P. aeruginosa* strains.

**Target group**		**Primer**					
**Accession**	**Name**	**F3**	**F2**	**F1c**	**B1c**	**B2**	**B3**
NC_017548.1	*Pseudomonas aeruginosa* M18	0	0	0	1	0	0
NC_020912.1	*Pseudomonas aeruginosa* B136-33	0	0	0	0	1	0
NC_008463.1	*Pseudomonas aeruginosa* UCBPP-PA14	0	0	0	0	0	0
NC_018080.1	*Pseudomonas aeruginosa* DK2	0	0	0	1	0	0
NC_011770.1	*Pseudomonas aeruginosa* LESB58	0	0	0	1	0	0
NC_009656.1	*Pseudomonas aeruginosa* PA7	0	0	1	1	0	0
NC_021577.1	*Pseudomonas aeruginosa* RP73	0	0	0	0	0	0
NC_017549.1	*Pseudomonas aeruginosa* NCGM2.S1	0	0	0	1	0	0
NC_002516.2	*Pseudomonas aeruginosa* PAO1	1	0	0	0	0	0

Table S17. The group-specific LAMP primer set designed by GLAPD for *P. aeruginosa* based on database-1.

Primer	Sequence(5'-3')
**F3**	TTTCGTTGTGCCGCCTTT
**B3**	CGAGGACGAAACGGTTGG
**FIP**	ATCGCGGAGAAGATCCACGC-TCTTTGGGGGATATCCGTGT
**BIP**	GAGCTGCCGTCCCAACAATTCA-GTGCATACACACGCAATTCG

2 Supplementary examples

Apart from *Staphylococcus aureus*, other bacteria were also used to compare GLAPD with other designers. The first example was *Helicobacter pylori*. There were 50 complete genomes of *H. pylori* in Database-1 and the existing LAMP primer set from Saori Horiuchi *et al.*[1] were not common to all 50 genomes (Table S14). Traditionally, the *cagA* gene was used to design primers for *H. pylori*, but less than 50% nucleotides of this gene were identical in each genome. When designing common primers for these 50 *H. pylori* genomes by PrimerExplorer and FastPCR based on *cagA* gene, no LAMP primer set could be designed successfully. However, when GLAPD took the 50 genomes as the target group and all other bacteria in Database-1 as the background group, it could design group-specific LAMP primer sets for *H. pylori* successfully. One primer set listed in Table S15 could amplify all the 50 genomes without any mismatches and it was specific to these 50 genomes only. The second example was *Pseudomonas aeruginosa*. There were 9 complete genomes of *P. aeruginosa* in Database-1, the existing LAMP primer set from Chao Li *et al.*[2] were not common to all 9 genomes (Table S16). *P. aeruginosa*-specific gene hypothetical protein (GenBank ID: 882161) was usually used to design primers. The sequences of this gene in different genomes were very similar. PrimerExplorer could design common primers using this gene, but there were many mutations in the designed primer regions in different target genomes (Figure S4, red shading). Unfortunately, FastPCR still could't design common primers using this gene. When GLAPD took the 9 genomes as the target group and all the other bacteria in Database-1 as the background group, it could design group-specific LAMP primer sets for *P. aeruginosa* successfully. One primer set listed in Table S17 could amplify all the 9 genomes without any mismatches and it was specific to these 9 genomes only.

Reference:

1. Horiuchi S, Nakano R, Nakano A, Hishiya N, Uno K, et al. (2019) Development of a loop-mediated isothermal amplification assay for rapid Helicobacter pylori detection. J Microbiol Methods 163: 105653.

2. Li C, Shi Y, Yang G, Xia XS, Mao X, et al. (2019) Establishment of loop-mediated isothermal amplification for rapid detection of Pseudomonas aeruginosa. Exp Ther Med 17: 131-136.
